# Clinicians’ Training Needs and Perceptions in Communicating Bad News in Emergency Health Care: A comparative study across rural and urban Health Care Units

**DOI:** 10.1192/j.eurpsy.2025.1861

**Published:** 2025-08-26

**Authors:** C. Pires-Lima, S. Morgado-Pereira, M. Figueiredo-Braga

**Affiliations:** 1 Neurosciences and Mental Health Department; 2 CINTESIS – Center for Health Technology and Services Research, Faculty of Medicine, University of Porto, Porto; 3 IVAR – Research Institute for Volcanology and Risk Assessment, University of the Azores, Ponta Delgada, Azores; 4 i3S – Institute for Research and Innovation in Health, University of Porto, Porto, Portugal

## Abstract

**Introduction:**

Communicating bad news is common in the health care sector, especially in emergency services. The importance of formal training in giving bad news in health care and the differences in the services provided by health care units in urban and rural contexts are well-documented in the literature. However, opportunities for clinicians to develop bad news communication skills provided by the distinct contexts of the health care unit in which they are included have rarely been studied. We assume that the communication of bad news is part of the service provided in emergency departments. Therefore, it can vary across rural and urban health care units.

**Objectives:**

This comparative cross-sectional study aims to test whether the location of the health care unit (rural vs. urban) has a significant impact on the communication of bad news by clinicians in emergency services.

**Methods:**

Data will be collected through an online questionnaire based on the literature in two purposive samples of emergency health care professionals in rural and urban contexts. Qualitative and quantitative methods will be applied to analyze data regarding work features, situational context, experience, perceived knowledge and skills in providing bad news, and training needs and preferences.

**Results:**

Rural health care units serve populations with more health disparities and poorer outcomes than non-rural, and classically have shortage of emergency medicine trained physicians. In urban areas health units have larger and more differentiated teams. The results will be discussed in the light of the literature on discrepancies between rural and urban health care units, describing professional characteristics and experience in delivering bad news of the study participants. We expect to identify specific contextual factors associated with geographic location, institutional settings, and health professionals’ training in delivering bad news in emergency medicine.

**Conclusions:**

The results of this study can aid to differentiate bad news communication trainers and health care unit managers in rural and urban areas (a) justifying the implementation of training programs, (b) adapting training programs to the audience, and (c) improving institutional facilities, practices, and policies to support adequate communication of bad news in emergency settings.

**Disclosure of Interest:**

None Declared

